# Heavy Metal and Trace Metal Analysis in Soil by Sequential Extraction: A Review of Procedures

**DOI:** 10.1155/2010/387803

**Published:** 2010-04-18

**Authors:** Amanda Jo Zimmerman, David C. Weindorf

**Affiliations:** ^1^Louisiana State University Geology and Geophysics, Baton Rouge, LA 70803, USA; ^2^Louisiana State University AgCenter, Baton Rouge, LA 70803, USA

## Abstract

Quantification of heavy and trace metal contamination in soil can be arduous, requiring the use of lengthy and intricate extraction procedures which may or may not give reliable results. Of the many procedures in publication, some are designed to operate within specific parameters while others are designed for more broad application. Most procedures have been modified since their inception which creates ambiguity as to which procedure is most acceptable in a given situation. For this study, the Tessier, Community Bureau of Reference (BCR), Short, Galán, and Geological Society of Canada (GCS) procedures were examined to clarify benefits and limitations of each. Modifications of the Tessier, BCR, and GCS procedures were also examined. The efficacy of these procedures is addressed by looking at the soils used in each procedure, the limitations, applications, and future of sequential extraction.

## 1. Introduction

Soils are the reservoir for many harmful constituents, elemental and biological, including heavy metals and trace metals, henceforth referred to as just metals [1]. Total metal content of soils is useful for many geochemical applications but often the speciation (bioavailability) of these metals is more of an interest agriculturally in terms of what is biologically extractable [2]. Speciation is defined by Tack and Verloo [3] as “the identification and quantification of the different, defined species, forms or phases in which an element occurs” and is essentially a function of the mineralogy and chemistry of the soil sample examined [4]. Quantification is typically done using chemical solutions of varying, but specific, strengths and reactivities to release metals from the different fractions of the examined soil [5]. In terms of bioavailability, various species of metals are more biologically available than others [6]. If bioavailability and the mobility of metals are related, then the higher the concentration of mobile toxic metals (Cu, Pb, Cd, and Al) in the soil column which increases the potential for plant uptake, and animal/human consumption [3, 7, 8]. 

Determination of metals in soil can be accomplished via single reagent leaching, ion exchange resins, and sequential extraction procedures (SEP), the latter under controversy. The number of available extraction techniques developed over the last three decades begs inquiry as to which technique is preferable over another. Moreover, the nonselectivity of the reagents used, handling of sediment prior to extraction, sediment-reagent ratio, and length of extraction all have an effect on data collected from SEP [3, 9] and can lead to inconsistent results even with the use of the same SEP. For true scientific collaboration to occur, a single SEP and set of standards would need to be adopted and applied across disciplines. The procedure adapted by Tessier et al. [4] is generally accepted as the most commonly used protocol followed closely by the BCR [5, 10, 11] but is still plagued by limitations discussed below.

This paper examines five SEP techniques recently referenced in the literature by comparing fractions, reagents used, and length of extraction. Modifications to these procedures are also discussed as are the soils used in each case, limitations to, and applications of the SEP.

## 2. Sequential Extraction Procedures

The theory behind SEP is that the most mobile metals are removed in the first fraction and continue in order of decreasing of mobility. All SEPs facilitate fractionation. Tessier et al. [4] named these fractions exchangeable, carbonate bound, Fe and Mn oxide bound, organic matter bound, and residual. These are also often referred to in the literature as exchangeable, weakly absorbed, hydrous-oxide bound, organic bound, and lattice material components, respectively [12]. Typically metals of anthropogenic inputs tend to reside in the first four fractions and metals found in the residual fraction are of natural occurrence in the parent rock [8]. 

The exchangeable fraction is removed by changing the ionic composition of water allowing metals sorbed to the exposed surfaces of sediment to be removed easily. A salt solution is commonly used to remove the exchangeable fraction. The carbonate-bound fraction is susceptible to changes in pH; an acid solution is used second. Metals bound to Fe and Mn oxides are particularly susceptible to anoxic (reducing) conditions so a solution capable of dissolving insoluble sulfide salts is used third. To remove metals bound in the organic phase, the organic material must be oxidized. The residual fraction consists of metals incorporated into the crystal structures of primary and secondary minerals. This fraction is the hardest to remove and requires the use of strong acids to break down silicate structures [4].

Most SEPs follow similar fractional degradation with little variation. Ure et al. [13] extracted the exchangeable and carbonate-bound fractions in a single step versus the two steps used in the Tessier procedure. The SEP used by the Geological Survey of Canada (GSC) divides the Fe and Mn oxide fractions into the amorphous oxyhydroxides and crystalline oxides, thereby increasing sequential fractionation from five to six steps [14]. Other SEPs with greater fractions include the procedure developed by Zeien and Brümmer [15] which included EDTA extractable, moderately reducible, and strongly reducible fractions for a total of seven; and that by Miller et al. [16] which consisted of nine fractions designed to test waste amended and agriculturally polluted sediments. 

The information needed from the SEP determines, to some extent, how the extraction is performed with respect to the final fraction, the residual. From a geochemical standpoint, total metal concentration is desired requiring the use of often dangerous reagents. From a biological or agricultural standpoint, less dangerous reagents may be utilized in lieu. The extraction conditions and reagents are listed in Table 1 for the five discussed SEPs. 

### 2.1. Tessier Procedure

In the extraction procedure by Tessier et al. [4], 1 g of sample is placed in a 50 mL tube. The sample is exposed to reagents and shaken (Table 1a). Each fraction is separated from the supernatant by centrifugation at 10,000 rpm, (~12,000 gravity) for 30 min. The supernatant is collected for lab analysis. The sediment is rinsed with 8 mL of deionized water (DIW) and centrifuged again. For the fourth fraction, a 1 g (dry weight) sample is exposed to 12 mL of 5 : 1 HF-HClO_4 _ acid mixture and evaporated to near dryness. A 10 : 1 HF-HClO_4_ acid mixture is added to the sample and again evaporated to near dryness followed by 1 mL of HClO_4_, evaporated until white fumes are visible. The final digestion is performed with 12 N HCl and diluted to 25 mL.

In the modified Tessier procedure, [17] analyzed two soils: one with moderate metal contamination and one with heavy contamination. The reagents stay the same but the amounts increase. Fraction one is run as normal. The reagent used in fraction two is increased from 8 mL to 50 mL, with continuous agitation for 5 hrs. The reagent used in fraction three for the heavily contaminated soil is also increased to 50 mL with continuous agitation for six hours. Fractions four and five remain unchanged. Rauret et al. [18] determined that an increase in the amount of reagent used increased the concentration of metals extracted for fractions two and three. They determined that the level/type of contamination of the tested sediment had a direct effect on the results obtained and by increasing the amount of used solution from 8 mL and 20 mL, respectively, to 50 mL, and were able to extract the maximum amount of metal without saturation.

### 2.2. Community Bureau of Reference (BCR) Procedure

This procedure is largely similar to that produced by Tessier et al. [4] with the chief difference in the first fraction of the procedure. Instead of evaluating the exchangeable and carbonate bound separately, the BCR procedure combines both in the first fraction [13]. 

 In the BCR procedure, 1 g of sample is placed into a 100 mL tube, exposed to reagents and shaken (see Table 1a). After each fraction, the solution is centrifuged at ~5000 rpm (3000 gravity) for 20 min and the supernatant is collected. The residue is washed in 20 mL of distilled water (D/W) for 15 min, and centrifuged. The residual fraction is not discussed in further detail and it is assumed that the steps closely follow those of Tessier [17, 19].

The BCR procedure was modified by a group of European experts in order to create an accepted protocol that could be used and the results easily reproduced. The modified procedure, again, is largely similar to the original. During fraction one, it was recommended that the sediment remains in suspension at all times during agitation. For fraction 2, the concentration of the reagent used is increased from 0.1 mol to 0.5 mol. The authors also recommended the addition of a fixed amount of concentrated HNO_3_, pH 1.5 during the making of the fraction 2 reagent [18].

### 2.3. Short Extraction Procedure by Maiz

Maiz et al. [12] conducted a comparison between the Short and Tessier procedures and found that the Short procedure produced strong correlation data for many metals tested. Three grams of residue are placed in a 50 mL tube, exposed to reagents and shaken (Table 1b). After the first extraction, the solution is centrifuged at 3000 rpm (~1000 gravity) for 10 min, the supernatant removed, and analyzed. The sample is then washed in 10 mL of bidistilled water and centrifuged. For the residual fraction, the residue is placed in teflon tubes with aqua regia—HF acid for an undetermined time [20].

### 2.4. Galán Procedure

This procedure is also similar in structure to the Tessier and BCR procedures. However, this procedure was used in extracting metals from soils severely affected by acid mine drainage in Spain such as those seen along the Rio Tinto [21]. Amorphous iron oxy-hydroxides can coat soils resulting is unobtainable data from regularly used techniques such as x-ray diffraction. Initial use of the Galán et al. [21] method showed increased accuracy of metals extracted in these soils than the Tessier and BCR methods.

 One-half a gram of soil sample is placed into tubes and exposed to reagents (Table 1b). Centrifugation of the sample, collection of the supernatant, washing, and fraction 4 are analyzed in the same manner as in the aforementioned Tessier extraction.

### 2.5. Geological Society of Canada (GCS) Procedure

One gram of sample is placed in a 50 mL tube and exposed to reagents and shaken (Table 1c). In between each fraction samples are centrifuged for 10 min at ~1000 g (2800 rpm). The supernatant is collected and the samples are washed in 5 mL of water, centrifuged, adding the wash water to the previous supernatant. Repeat the washing procedure. Prior to performing the fourth fraction (Table 1c). The amount of time needed to complete the fraction is proportional to the time for the reduction of sample to an appropriate volume. 

The modified GCS is the most modified of the SEPs. The run time is drastically shortened and the reagents changed. Table 2 depicts these changes. Benitez and Dubois [22] modified the GCS procedure by testing various reagents at various time in varying order. They determined that no one sequence of events were fully satisfactory for a SEP but recommended one particular method above the others. That experiment was later adapted by D*œ*lsch et al. [23] into the modified GCS.

## 3. Soils

The soil used by Tessier et al. [4] in developing their SEP was not characterized beyond identification of a bottom soil. However, the subsequent modification by Rauret et al. [17] focused on soils characterized as mildly contaminated and heavily contaminated. Since the Tessier procedure was developed to extract metals Cd, Co, Cu, Ni, Pb, Zn, Fe, and Mn the use of this method for soils exposed to large anthropological inputs is obvious. The Tessier SEP can be used on a broad array of soil types provided the metals tested for are those listed above. This is probably why the Tessier SEP is the most used procedure to date [5].

In the original study by Ure et al. [13] to produce the BCR SEP four labs were sent batches of seven sediments and one sewage amended soil to analyze by multiple extraction methods with the final results meant to determine best extractional procedures for each fraction as well as the “recipe” for certified reference sediments. Little to no discussion of the actual sediments and soil are given beyond this; but in the modification study [18] the researchers discussed the use of CRM 601 a reference material designed to have specifically extractable components. This allowed for the research team to verify the viability of proposed modifications. Rodríguez et al. [24] used this procedure on sixty soil samples exposed to Zn-Pb mining activities and surrounding croplands in Spain. Metal contaminations of Pb, Zn, Cd, and Cu were identified using this procedure in tailings from the mine as well as soil at distance from the tailings indicating movement of metal contamination with time via water or wind transports. Hossain et al. [25] used the original BCR procedure and isotherm fitting to characterize metals on surface soils overlaying a typical loam soil in the Kanto plains, Japan. Through analysis they determined that the surface contained greater amounts of humic material while the lower soil was more dominant in carbonate material. Using isotherm fitting data they also determined that competition effects of heavy metals produced an effect on the mobility of other metals in the column. Meaning the sorbtive behavior of Zn would decrease with increasing amounts of Ni or Cu in both the surface and underlying soils for example. The same trend was evident for Ni with increasing levels of Zn or Cu. 

 The short SEP was tested against the Tessier and BCR SEPs on three soils contaminated by mining activities, steel factory smelting activities, and traffic emissions from Spain. The samples were collected from the top ten centimeters of the column. All three collection locations were known to have high concentrations of Pb, Zn and other metals such as Cd, Cr, Cu, Mn, Ni, and Fe. The authors were not able to directly compare the results because of the differences in reagents used but did determine all procedures produced the same order of extraction: Cd > Pb > Zn *≈* Cu > Mn > Ni > Fe *≈* Cr. 

 Galán et al. [21] also used soil heavily contaminated by acid mine drainage related to Río Tinto and Río Odiel. However, these soils contained a poorly crystalline Fe oxy-hydroxide coatings that made other kinds of analysis difficult. The goal was to remove the resistant poorly crystalline coatings to prepare the samples for x-ray diffraction using hydroxylamine hydrochloride. 

 In the development of the GCS SEP ten different certified reference samples were tested, SRM 2709–2711, SO-1–4 series, MAG-1 a marine mud, LKSD-4 lake sediment, and TILL-2 a till sample. The researchers examined for twenty different elements and compared the results to those published for the said reference materials. This method produced good correlation to the published data excluding that for Cr and V which require an extra step for dissolution. During modification of this method, Benitez and Dubois [22] used three different soils from the Swiss Jura region, a calcaric rich sample, a sample with a high clay content, and a soil rich in organic matter in order to determine the best method for Cd speciation. By using the different soil types they were able to isolate which reagent was better suited to each fraction and in what order extraction should occur and though no one procedure stood out above the other two the modified GCS did provide the most realistic results. Dœlsch et al. [23] used the modified GCS and BCR procedures to characterize tropical volcanic soils of Réunion island. The two methods produced good agreement in extraction results except in the fraction bound to organic matter in which the BCR procedure underestimated the level of metals bound while the GCS procedure did not.

## 4. Problems and Limitations of the SEP

As long as SEPs have been around there has been controversy over the nonselectivity of the reagents, which may alter surface chemical characteristics of sediments tested, and potential for metals to redistribute among the remaining fractions during the extraction process by sorbing to the freshly exposed surfaces [10, 11]. Studies have employed model soils composed of natural mineral and humic acid, or the use of standard addition by adding a pure synthetic component to real sediments prior to extraction. Model soils used by Shan andChen[10] indicated that redistribution was in fact occurring. Metals collected for fractions 1–3 were less than should have been and for fractions 4-5 were greater. This was a direct indication that as metals were released in the first three fractions they were reattaching to the newly available sites of the next fraction. Metals Cu, Mn, Ni, Pb, V and Zn were collected primarily from the fourth fraction due to the strong complexes these metals tend to form with humic material. Both soil composition and the nature of the metal played a large part in the amount of redistribution that occurred due to different binding sites available and varying binding strengths.

XRD is also a useful tool in characterizing reactivity of silicate clays during the extraction process. Ryan et al. [5] examined samples before extraction, between each phase of extraction and after the extraction was complete to determine if any changes occurred to the soil directly because of the extraction process. They determined that destruction of the octahedral sheet of trioctahedral clays was evident with octahedral Mg-O bonds quite vulnerable to hydrolysis. The significance being that during the first three phases of extraction metals being released do not comprise just the fractions for which they are designed, but can also release metals in structural sites thus skewing results on true bioavailability.

This is complemented by a study also utilizing XRD after the fourth, fifth, and sixth fractions of an extraction procedure adapted after Tessier's five step procedure [26]. The mineralogy of the sampled soils varies significantly in a short distance which has an effect on the total metals determined from each sample. They were able to determine metal type that was the main factor controlling the distribution of metals in the Szklary region, Poland.

## 5. Conclusions

There are many SEPs that can be utilized in the process of understanding the behavior of metals in various soils. The researcher trying to determine which procedure is most appropriate for their samples must take into consideration many factors including soil type, contamination level, and result comparison methods, as well as the potential problems or limitations associated with a specific SEP. 

Is it clear that reliance on the SEP alone is not feasible and needs to be complemented with either XRD analysis or some other kind of analytical technique to positively identify the solid components involved. This will provide enough data to make a better calculated determination on the amounts of metals in a soil as well as their potential speciation. However, because of the evidence for redistribution during fractionation, competition among various metals, and the nonselectivity of reagents for each fraction, it may better suit future studies using SEPs do so with the understanding that the fractional quantification will be skewed toward lower than real results for the fractions related to exchange, carbonate bound and reducible bound metals and skewed toward higher than real results for organic bound and residual metals. 

The future of the SEP is not as bright as once believed but is still useful. An understanding of the behavior of metal contaminants at various biologically available fractions is still necessary especially when human consumption is becoming more of a global concern with the current growth rate of populations, especially in urban settings. More work needs to be done on improving the specificity of reagents and with combining SEP data with analytical data such as that obtained via XRD.

## Figures and Tables

**Table:** (a)

	Time	Temp	Quantity	Tessier	Time	Temp	Quantity	BCR
				1 g				1 g
Exchangeable	1 hr	continuous agitation	8 mL	1 mol MgCl_2_ pH 7.0	16 hr	22°C ± 5° w/constant agitation	40 mL	0.11 mol CH_3_COOH
			8 mL	or 1 mol Na OAc pH 8.2				
Bound to Carbonates	5 hr	continuous agitation-leached at rm temp.	8 mL	1 mol Na OAc pH 5.0 w/acetic acid				
Bound to Iron and Manganese Oxides	6 hr		20 mL	0.3 mol Na_2_S_2_O_4 _+ 0.175 mol Na-citrate + 0.025 mol H-citrate	16 hr	22°C + 5° w/constant agitation	40 mL	0.1 mol NH_2_OH_∗_HCl pH 2 with HNO_3_
		or 96° ± 3 occasional agitation	20 mL	0.04 mol NH_2_OH*HCl in 25% (v/v) HOAc				

Bound to Organic Matter	2 hr	85° ± 2 with occasional agitation	3 mL	0.02 mol HNO_3_	1 hr	room temp. w/occ. Agitation	10 mL	8.8 mol H_2_O_2_ pH 2-3
			5 mL	30% H_2_O_2_ pH 2 with HNO_3_				
	3 hr	85° ± 2 with intermittent agitation	3 mL	30% H_2_O_2_ ph 2 with HNO_3_	1 hr	85° degrees C	10 mL	reduce vol. to less than 3 mL H_2_O_2_ pH 2-3 reduce vol. to 1 mL
					1 hr			
	30 min	continuous agitation	5 mL	3.2 mol NH_4_OAc in 20% (v/v) HNO_3_-dilute to 20 mL	16 hr	22°C + 5° w/constant agitation	50 mL	1 mol NH_4_OAc pH 2 w/HNO_3_

Residual			1 mL Unk	HF-HClO_4_ 5 : 1 HF-HClO_4_ 10 : 1 HClO_4_ 12 N HCl				HF, HNO_3_, HClO_4_

**Table:** (b)

	Time	Temp	Quantity	Maiz-Short	Time	Temp	Quantity	Galán
				3 g				0.5 g
Exchangeable		rm temp.	10 mL	0.01 mol CaCl_2_		20°C w/continuous agitation	35 mL	1 M NH_4_OAc, pH 5
	2 hr	suspend under agitation			1 hr			
Bound to Carbonates	4 hr			0.005 mol DTPA +				
Bound to Iron and Manganese Oxides		rm temp.		0.01 mol CaCl_2_ +		96°C manual agitation every 30 min	20 mL	0.4 M NH_2_OH*HCl in CH_3_COOH 25%
				

		2 mL	0.1 mol TEA pH 7.3	6 hr
Bound to Organic Matter					2 hr	85°C w/manual agitation every 30 min	3 mL	0.2 M HNO_3_
							5 mL	30% H_2_O_2_, pH 2
					3 hr		3 mL	30% H_2_O_2_
					30 min	Continuous agitation	5 mL	30% H_2_O_2_
Residual				*aqua regia*-HF acid	2 hr		10 mL	HF,HNO_3_,HCl 10 : 3 : 1

**Table:** (c)

			Time	Temp	Quantity	Canada
						1 g
Exchangeable	mobile	AEC	6 hr		20 mL	1.0 mol CH_3_CO_2_Na pH 5
			6 hr		20 mL	1.0 mol CH_3_CO_2_Na pH 5
Bound to Carbonates		Am Fe ox	2 hr	60°C vortex every 30 min	20 mL	20 mL 0.25 mol NH_2_OH_∗_HCl in 0.05 mol HCl
Bound to Iron and Manganese Oxides			30 min	60°C	20 mL	20 mL 0.25 mol NH_2_OH_∗_HCl in 0.05 mol HCl
		Cry Fe ox	3 hr	90°C vortex every 20 min	30 mL	1.0 mol NH_2_OH_∗_HCl in 25% CH_3_CO_2_H
Bound to Organic Matter	mobilisible		1.5 hr	90°C	30 mL	1.0 mol NH_2_OH_∗_HCl in 25% CH_3_CO_2_H
		Org/Sulf	30 min			750 mg KClO_3_ and 5 mL 12 mol HCl vortex and add 10 mL HCl more
						15 mL H_2_O
			20 min	90°C	10 mL	4 mol HNO_3_

Residual		silicates and residual	Unk	200°C	2 mL	16 mol HNO_3_~reduce to 0.5 mL
			20 min	90°C	2 mL	12 mol HCl
			1 hr	90°C	10 mL	acid mix H
			overnight	Evap 70°C		
			last bit	Rai 120°C		
			5–10 min		1 mL	12 mol HCl
					3 mL	16 mol HNO_3_
					3 mL	3 mL H_2_O and warm then bring up to 20 mL

**Table 2:** Operating conditions for the modified GCS extraction procedures using 1 g of sample.

	Time	Temp	Quantity	Canada
				Benitez and Dubois [22]
Exchangeable	1.5 hrs	25°C	30 mL	0.1 mol NaNO_3_
	1.5 hrs	25°C	30 mL	0.1 mol NaNO_3_
Adsorbed	1.5 hrs	25°C	30 mL	1 mol Na OAc pH 5.0 w/CH_3_COOH
	1.5 hrs	25°C	30 mL	1 M Na OAc pH 5.0 w/CH_3_COOH
Organic	1.5 hrs	25°C	30 mL	0.1 mol Na_4_P_2_O_7_
	1.5 hrs	25°C	30 mL	0.1 mol Na_4_P_2_O_7_
Amorphous Oxyhydroxides	1.5 hrs	60°C	30 mL	0.25 mol NH_2_OH_∗_HCl in 0.5 mol HCl
	1.5 hrs	60°C	30 mL	0.25 mol NH_2_OH_∗_HCl in 0.5 mol HCl
Crystalline Oxides	1.5 hrs	90°C	30 mL	1 mol NH_2_OH_∗_HCl in 25% H Oac
	1.5 hrs	90°C	30 mL	1 mol NH_2_OH_∗_HCl in 25% H Oac
Residual	unk			HF, HNO_3_, HClO_4_
